# Double outlet right ventricle, pulmonary stenosis and congenital mitral stenosis

**DOI:** 10.1007/s12055-019-00896-x

**Published:** 2020-01-08

**Authors:** Lincoln Samuel, George Varghese Kurien, Joel Devasia Vazhakatt, Sajan Koshy

**Affiliations:** 1 Paediatric Cardiac Surgery, Aster Medcity, Kochi, 682027 India; 2 Cardiovascular & Thoracic Surgery, Aster Medcity, Kochi, 682027 India; 3 Cardiac Anaesthesia & Critical Care, Aster Medcity, Kochi, 682027 India; 4 Paediatric Cardiac Surgery, Aster Medcity, Kochi, 682027 India

**Keywords:** Tetralogy of Fallot (TOF), Supramitral ring, Parachute mitral valve

## Abstract

A 4-year-old girl child was diagnosed with double outlet right ventricle (DORV), severe pulmonary stenosis, and supramitral ring. This case is presented to bring to light this rare association. Through this report, we aim to stress importance of assessing mitral apparatus on echocardiography during evaluation for situations like DORV and Tetralogy of Fallot (TOF). The physiological differences in such situations as opposed to their isolated counterparts and special postoperative outcomes are also discussed.

## Introduction

TOF, since its first published description in 1888, is known to be associated with a myriad of other cardiac anomalies. Of these, the least described and sometimes missed are left-sided heart lesions, particularly congenital mitral stenosis. While concentrating on the four classical components of tetralogy, its association with other cardiac defects may go unnoticed.

## Case report

A 4-year-old girl child presented with severe cyanosis. She was diagnosed with complex congenital heart disease at 1.5 years of age. At presentation, she weighed 12.5 kg, with an oxygen saturation of 74% on room air. She had recurrent cyanotic spells triggered by activity, relieved by squatting, for the last 3 years. 2D echocardiography done at our hospital revealed DORV (tetralogy like), moderate infundibular stenosis causing right ventricular outlet tract obstruction with pulmonary annulus of 13 mm (*z*-score − 0.59), severe mitral stenosis with parachute mitral valve and supra mitral ring (SMR) with an effective mitral orifice of 10 mm (*z*-score − 1.87), both atrial chambers and right ventricle being dilated. Pre-operative mitral valve gradients were maximum of 23 mmHg and mean of 9.86 mmHg, and the pulmonary gradient was 50 mmHg. Chest X-ray showed cardiomegaly with an upturned apex and pulmonary venous hypertension.

**Fig. 1:**
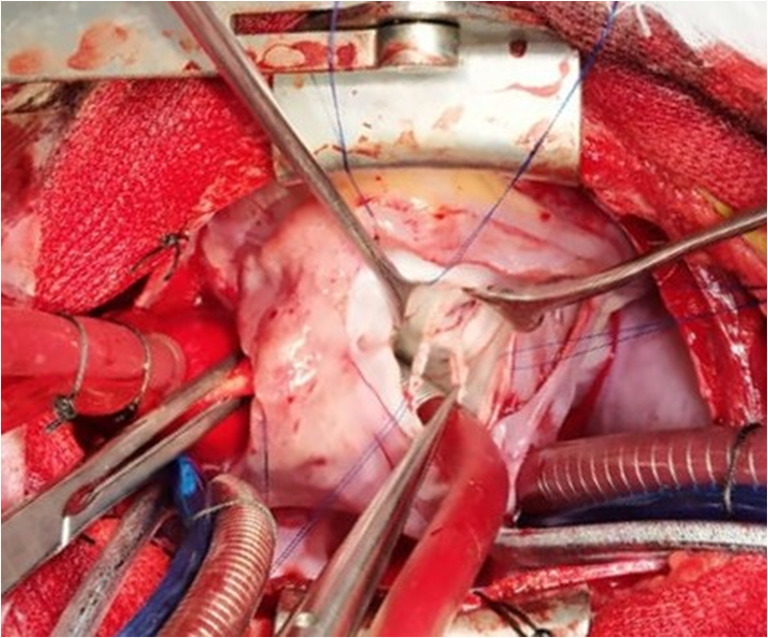
Supramitral ring being excised, forceps points towards partly excised ring.

We undertook an elective intracardiac repair with excision of the SMR. A 15-mm subaortic ventricular septal defect and DORV with right ventricular outflow tract obstruction (RVOTO) were present. There was a complete supra mitral ring, 3 mm above the mitral annulus, narrowing the atrio-ventricular inflow (Fig. 1). The valve apparatus was the classical parachute mitral valve with both leaflets arising from a single papillary muscle. Anterior and posterior chordal groups were sufficiently spaced, with no evidence of significant subvalvar stenosis.

Following SMR excision, mitral orifice was admitting a size 14 Hegar’s dilator. There was no valve incompetence. The ventricular septal defect (VSD) closure was done through the right atrium using a poly-tetraflouro-ethylene patch routing the aorta to the left ventricle (LV). An incision was made proximal to a conal branch of coronary artery, crossing the RVOT. The obstructing infundibular muscle bands were resected. The RVOT was widened with a patch of gluteraldehyde-treated autologous pericardium. The pulmonary valve leaflets appeared normal in morphology and the outlet admitted a size 18 Hegar’s dilator. The child had stable sinus rhythm after discontinuing heart-lung machine support.

She was successfully extubated on the 1st postoperative day and discharged by the 8th day. Postoperative echocardiography showed improvement in the mitral orifice to 14 mm (*z*-score − 1.02) with maximum gradient of 5.25 mmHg and mean of 2.49 mm5Hg. Pulmonary gradient was 28 mmHg.

At 1-month review, the echo parameters were the same as the immediate postoperative period. Cyanosis had disappeared and she had significant improvement in exercise tolerance.

## Discussion

Fisher first described isolated stenotic SMR as early as 1908. J. D. Shone described parachute mitral valve in 1963 [1]. SMR associated with other congenital mitral lesions is known to cause death in nearly half of affected children in the first year of life [1].

The earliest described association of congenital mitral stenosis and tetralogy are by Hohn et al. (1968) [2] and Benry, Leachman (1976) [3]. They reported cases where the SMR in association with Tetralogy of Fallot were found at post mortem in patients who had undergone intracardiac repair. The incidence of this association is however small. In a large (> 2200 patients) single-center series, Changela and colleagues published a 0.4% association of mitral valve disease with tetralogy in 2010 [4].

**Fig. 2:**
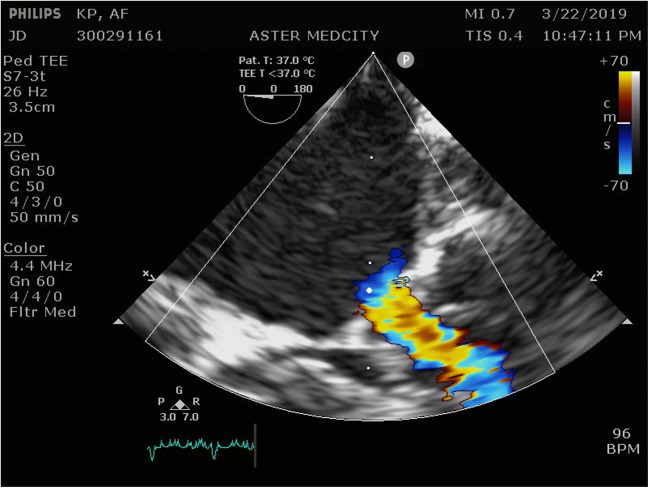
Polar light sign in echocardiogram

The association of left- and right-sided low flow as in this case raises several concerns in the management of such patients. In the setting of pulmonary stenosis, presence of mitral stenosis potentiates pulmonary venous congestion leading to hypoxemia [1]. Mitral stenosis may predispose the patient to atrial fibrillation, though not common in Tetralogy like situations [1]. Tetralogy is already a low flow situation. In this setting, presence of mitral stenosis will further reduce the end diastolic volume, a known risk factor for early death after repair [1]. There is a high probability of missing out mitral valve lesions in patients with tetralogy on echocardiogram [5]. The classical echo finding in supramitral ring is a “polar light sign” appearance [5] (Fig. 2). In TOF patients, while detailed attention is paid to the anterior displaced ventricular septum and the RVOT obstruction, failure to evaluate the mitral valve can be devastating [5]. Presence of untreated mitral stenosis following intracardiac repair (ICR) will ultimately lead to poor postoperative outcomes including death.

Another key concern in the management of this particular patient was the parachute mitral valve in addition to the SMR. In this child, subvalvar apparatus (chordae and single papillary muscle) was not in any way restricting mitral inflow. However, the SMR would be the most probable factor preventing growth of the mitral annulus by restricting the flow across the valve.

We believe that improvement of right ventricular outflow and mitral inflow will allow the mitral annulus to grow. She would require close follow-up to assess the mitral valve. This report, also underlines the importance of thorough evaluation of the mitral valve apparatus in every case of TOF. Two stenotic lesions in the left ventricular inflow occurring along with TOF is a unique physiology. If the subvalvar obstruction is not significant, the mitral valve can be expected to grow upon release of SMR.

## References

[1] Kirklin JW. Baratt Boyes Cardiac Surgery. 4th Edition. Philadelphia: Churchill Livingstone;1814. Vol 2. 2003. 946-1073.

[2] Hohn AR, Jain KK, Tamer DM. Supravalvular mitral stenosis in a patient with tetralogy of Fallot. Am J Cardiol. 1968;22:733–7. 10.1016/0002-9149(68)90213-0 5683430

[3] Benry J, Leachman RD, Cooley DA, Klima T, Lufschanowski R. Supravalvular mitral stenosis associated with tetralogy of Fallot. Am J Cardiol. 1976;37:111–4. 10.1016/0002-9149(76)90509-9 1244728

[4] Changela V, John C, Maheshwari S (2010). Unusual cardiac associations with tetralogy of Fallot – a descriptive study. Pediatr Cardiol..

[5] Barik R, Nemani L. Tetralogy of Fallot associated with supramitral ring: “Paying for a near miss”. J Cardiol Cases. 2015;12:159–61. 10.1016/j.jccase.2015.06.008 PMC6281841 30546584

